# Effects of *Epimedium* Ultrafine Powder on Seminal Quality, Hormones, Immuno-Antioxidant Status, Gut Microbiota and Metabolites in Boars

**DOI:** 10.3390/ani16101520

**Published:** 2026-05-15

**Authors:** Jingbin He, Weiyi Li, Bin Ran, Yupeng Zhang, Junjie Wu, Yunxiang Zhao, Zhili Li, Mengjie Liu

**Affiliations:** 1 School of Animal Science and Technology, Foshan University, Foshan 528225, China; 2 College of Animal Science and Technology, Guangxi University, Nanning 530004, China; 3 Guangxi Yangxiang Agricultural and Animal Husbandry Co., Ltd., Guigang 537100, China

**Keywords:** ultrafine *Epimedium* powder, seminal quality, antioxidant, intestinal metabolites

## Abstract

*Epimedium* is a traditional Chinese tonic used to tonify the kidneys, enhance sexual function, and strengthen muscles and bones. However, the potential effects of *Epimedium* on the semen quality of Bama boars remain incompletely elucidated. The objective of this study was to evaluate the effects of dietary *Epimedium* ultrafine powder (EP) supplementation on the semen quality of Bama boars and to explore the underlying mechanisms. The objective of this study was to evaluate the effects of dietary EP supplementation on the semen quality of Bama boars and to explore the underlying mechanisms. Eighteen healthy, sexually mature adult male Bama boars were randomly divided into three groups (*n* = 6) and fed either a basal diet (CON) or the basal diet supplemented with 0.3% (EP3) or 0.5% (EP5) *Epimedium* ultrafine powder for five weeks. This study employed enzyme-linked immunosorbent assay (ELISA), 16S RNA gene sequencing, non-targeted metabolomics (CON and EP5), and Spearman correlation analysis, among other methods. The results indicated that dietary *Epimedium* (0.3% and 0.5%) increased the levels of serum TP, FSH, and SOD and decreased the abnormal sperm rate and the levels of serum TBA, TNF-α, and IL-6. Among them, adding 0.5% *Epimedium* in the diet increased sperm motility and the levels of serum T, LH, and IgG. 16S rRNA gene sequencing analysis revealed that both 0.3% and 0.5% *Epimedium* supplementation reduced the abundance of Streptococcus. Specifically, the 0.3% dose decreased *Prevotella* abundance, while the 0.5% dose reduced *Escherichia-Shigella* abundance. PICRUSt2 analysis revealed that the pathways of phenylalanine, butanoate, biotin, and arachidonic acid metabolism were significantly enriched in the *Epimedium* group. A non-targeted metabolomics analysis identified that indole-3-acrylic acid, DL-tryptophan, 2-hydroxyphenylalanine, and propionylcarnitine showed significant upregulation after *Epimedium* supplementation. Spearman correlation analysis indicated that *Streptococcus* was negatively correlated with sperm motility and serum-related parameters (TP, T, LH, IgM, and IgG). *Streptococcus* and *Escherichia-Shigella* were negatively correlated with indole-3-acrylic acid, DL-tryptophan, and biotin. In conclusion, *Epimedium* has a positive impact on the seminal quality, reproductive hormones, and immune–antioxidant levels of Bama boars by regulating the composition and metabolites of the intestinal microbiota.

## Simple Summary

*Epimedium* is a natural herbal supplement that has the effects of improving sexual function, regulating hormone levels, enhancing immunity, and antioxidant capacity. Supplementing the diet of Bama boars with *Epimedium* (0.5%) improved sperm motility and upregulated serum T, FSH, LH, IgG, and SOD levels, while simultaneously diminishing the abnormal sperm rate and serum TNF-α and IL-6 concentrations. Dietary *Epimedium* (0.5%) supplementation reduced the relative abundance of potentially pathogenic bacteria, such as *Streptococcus* and *Escherichia-Shigella*, while concurrently elevating the levels of key metabolites, including indole-3-acrylate and DL-tryptophan. These findings suggest that dietary *Epimedium* supplementation improves semen quality in Bama boars by coordinately modulating gut microbiota and metabolic profiles and optimizing serum biochemical parameters, thus supporting its potential application in reproductive health management.

## 1. Introduction

Reproductive biotechnologies, particularly Artificial Insemination (AI), serve as the cornerstone of genetic improvement strategies in swine and other livestock species [1]. However, their effectiveness depends largely on semen quality. Phytogenic feed additives are being increasingly used to enhance the reproductive efficiency and production of domestic animals due to their low toxicity and the effects of antioxidants and anti-inflammatories [2]. The research focus is also on seeking alternative phytogenic feed additives to enhance the reproductive performance of boars [3]. Studies have demonstrated that the dietary supplementation of boars with 7 g of Turnera diffusa extract affects both ejaculate volume and post-storage viability (48 h) [4]. Supplementation with *Epimedium* polysaccharide (EPS) improves goat semen characteristics following cryopreservation and substantially enhances sperm fertilizing capacity [5]. Previous studies have indicated that dietary supplementation with wolfberry (*Lycium barbarum*) results in varying degrees of improvement in boar semen quality, specifically regarding sperm progressive motility, total abnormality rate, sperm concentration, and total sperm count per ejaculate [6]. These additives modulate the metabolic and physiological processes of spermatogenesis, thereby enhancing semen utilization efficiency across the boar population.

As an indigenous Chinese breed, the Bamaxiang pig occupies a unique niche among native purebred swine populations. It is characterized by its compact stature and distinctive ‘two-ended black’ coat pattern [7]. Compared to other breeds, the Bamaxiang reaches puberty earlier and demonstrates superior attributes, including exceptional meat quality, robust adaptability, and heightened disease resistance [8]. However, in actual production, Bama boars may exhibit low sexual desire and declining reproductive performance, which compromises production efficiency. Therefore, improving semen quality is crucial not only for the protection and utilization of the breed but also for promoting the continuous optimization of superior pig breeds.

Herba epimedii (*H. epimedii*), a renowned traditional Chinese medicine, has been extensively utilized for centuries to improve male potency and treat erectile dysfunction [9]. Icariin (ICA), the main bioactive flavonoid glycoside in *Epimedium*, exhibits well-documented antioxidant, immunomodulatory, and anti-inflammatory effects [10]. A novel herbal formula combining *Epimedium* and *Angelica sinensis* (Danggui) significantly increased sperm production by mitigating oxidative stress, thereby demonstrating therapeutic efficacy in a male infertility model [11]. Icariin was employed as a feed additive to enhance reproductive performance in bucks (*Capra hircus*) by elevating serum hormone concentrations and boosting testicular antioxidant capacity [12]. Recent findings reveal an intricate interplay between gut homeostasis and systemic physiological functions, exerting significant modulatory effects on reproductive performance [13]. Studies have shown that the gut microbiota regulates the testicular microenvironment and sperm production through the gut–testis axis [14]. Numerous studies have highlighted the dual impact of gut microbiota (GM) on semen quality [15]. Beneficial taxa, such as *Lactobacillus*, *Bacteroidetes*, and *Ruminococcus* (UCG-011), promote spermatogenesis and enhance sperm motility [14]. Conversely, gut dysbiosis impairs sperm production and compromises motility [15]. This phenomenon is primarily attributed to gut microbiota dysbiosis, which exacerbates inflammation and disrupts oxidative stress-related enzyme activities, testosterone levels, and blood–testis barrier (BTB) permeability [16]. Although *Epimedium* is beneficial for male potency, the research on its beneficial effects on a native Chinese pig breed of Bama boars is still limited. Therefore, the direct effects of dietary ultrafine *Epimedium* powder on the sperm quality of Bama boars were elucidated by evaluating parameters associated with reproductive performance. We hypothesized that ultrafine *Epimedium* powder would enhance seminal quality not only by directly optimizing serum parameters (including reproductive hormones, antioxidant capacity, and immune levels) but also potentially through modulating the gut microbiota and metabolites. These findings will provide a certain research basis for future research on the gut microbiota–reproductive axis.

## 2. Materials and Methods

### 2.1. Animals, Diet, and Experimental Design

A total of 18 healthy and sexually mature Bama boars (8 months old) were selected and randomly assigned to 3 groups, with 6 boars in each group. The Bama boars are provided by Bama Original Breed Pig Farming and Industrial Co., Ltd. (Hechi, China). The boars were fed either a basal diet (control), a basal diet supplemented with 0.3% *Epimedium*, or a basal diet supplemented with 0.5% *Epimedium*. *Epimedium* ultrafine powder was procured from Guangdong Benyuan Technology Development Co., Ltd. (Foshan, China). In brief, dried *Epimedium* herbs were dried in a constant-temperature oven at 55 °C, pulverized, and sieved through a 60-mesh screen to obtain standard powder. For the preparation of ultrafine powder, the material was subjected to low-temperature airflow grinding (−20 °C) and passed through a 300-mesh screen, yielding particles with a median diameter (D50) of ≤10 μm. The major constituents of *Epimedium* extract were icariin (23.4%), epimedin A (2.82%), epimedin B (7.45%), epimedin C (11.65%), and baohuoside I (0.77%) [17]. The boars were individually housed under a 2.2 m-by-0.6 m enclosure for a 5-week trial. Boars were kept under controlled environmental conditions (20 ± 3 °C, 60–75% humidity), had free access to water, and were fed twice daily at 07:00 and 14:00. Table 1 presents the ingredients and nutrient composition of the basal diets. Semen samples were collected at the end of the trial using the gloved-hand technique. Following the protocol described by Gao et al. [18], four key semen parameters were assessed: volume, sperm concentration, motility, and abnormality rate. Plasma was separated by centrifugation (3000× *g*, 10 min) and stored at −80 °C. Fresh fecal samples were collected directly from the rectum following digital massage to stimulate defecation. Samples for 16S rRNA gene sequencing were snap-frozen in liquid nitrogen and kept at −80 °C.

### 2.2. Using a Computer-Assisted Sperm Analysis System to Detect Sperm Parameters

The sperm parameters, including sperm concentration, sperm motility, abnormal sperm rate, and sperm volume, were analyzed by a computer-assisted sperm analysis (CASAII) system (Shanghai Kasu Biotechnology Co., Ltd., Shanghai, China).

### 2.3. Detection of Serum Biochemical Variables and Antioxidant Capacity

Serum biochemical parameters, including alanine aminotransferase (ALT), aspartate aminotransferase (AST), TBA, total protein (TP), and albumin (ALB), were measured using a Mindray BS-380 fully automated biochemistry analyzer (Shenzhen Mindray Bio-Medical Electronics Co., Ltd., Shenzhen, China). The activities of antioxidant-related enzymes in serum, including the levels of GSH-Px (colorimetric method), SOD (WST-1 method), and T-AOC (FRAP method), were determined by kits purchased from Nanjing Jiancheng Bioengineering Institute (Nanjing, China).

### 2.4. Detection of Reproductive Hormones and Immune-Related Indicator Content

Based on previous studies [19], the indicators of the serum samples were analyzed using the ELISA kit. The concentrations of the immunoglobulin A (IgA), IgG, IgM, TNF-α, IFN-γ, interleukin (IL)-6, and reproductive hormones (T, LH, and FSH) in serum were measured using an enzyme-linked immunosorbent assay (ELISA) kit (Meimian Biotechnology, Nanjing, China).

### 2.5. Bama Boar Fecal Microbiota Sequencing

Fecal gut microbiota composition was profiled using bacterial 16S rRNA gene sequencing, as previously described [20]. Total genomic DNA was extracted from fecal samples of each Bama boar group using the FastPure Stool DNA Isolation Kit (QIAGEN, Hilden, Germany). Genomic DNA integrity was verified by agarose gel electrophoresis (1%). The bacterial 16S rRNA V3–V4 (338F: CCTAYGGGRBGCASCAG; 806R: GGACTACNNGGGTATCTAAT) regions were then amplified via PCR using target-specific primers. Following amplification, PCR products were purified and quantified. Sequencing libraries were prepared using the NEXTFLEX^®^ Rapid DNA-Seq Kit and sequenced on the Illumina NovaSeq 6000 platform. Bioinformatics analysis of the intestinal microbiota was performed following the microbiome profiling protocol reported by Liu et al. [21]. Alpha diversity was assessed using the Chao1, observed species, Shannon, and Simpson indices. Beta diversity was evaluated based on Bray–Curtis distance matrices. PCoA and PERMANOVA were performed to visualize and statistically test community differences. The nonparametric rank sum test was used to detect differences in microbial communities between groups. Taxonomic assignment was conducted using the SILVA 138.1 database. LEfSe analysis identified differentially abundant taxa across taxonomic ranks from phylum to genus (LDA > 4.0). Intergroup differences were detected using the nonparametric rank-sum test, while Spearman’s rank correlation analyzed species associations.

### 2.6. Metabolomics Analysis of Bama Boar Fecal Samples

Fecal samples (*n* = 5) from the CON and EP5 groups were submitted to Novogene Bioinformatics Co., Ltd. (Tianjin, China) for untargeted metabolomics analysis. Metabolites were extracted using 400 μL of methanol: water (4:1, *v*/*v*) containing L-2-chlorophenylalanine (0.02 mg/mL) as the internal standard. The extracts were analyzed by LC-MS/MS using a Thermo Scientific LTQ XL mass spectrometer (Thermo Fisher Scientific, Waltham, MA, USA). Data processing, including peak picking, alignment, and quantification, was conducted using Compound Discoverer 3.1 software (Thermo Fisher, USA). Metabolite identification was performed by matching spectra against the HMDB, METLIN, and LIPID MAPS databases. Differential metabolites were screened using PCA and PLS-DA models, with selection criteria of VIP > 1 and *p* < 0.05. Finally, KEGG-based enrichment analysis was utilized to determine the pathways associated with the identified differential metabolites.

### 2.7. Statistical Analysis

Data were compiled in Microsoft Excel and analyzed using SPSS 26.0 and GraphPad Prism 8.5. All data were subjected to normality and homogeneity of variance tests, and one-way analysis of variance (One-way ANOVA) was employed. Duncan’s method was used for multiple comparison analysis of differences among groups. All data were expressed as mean  ±  standard deviation (SD). Statistical significance was defined as *p* < 0.05.

## 3. Results

### 3.1. Effect of Dietary Epimedium on Semen Quality of Bama Boars

As indicated in Table 2, the rate of sperm abnormalities in the Bama boars was lower in the EP3 and EP5 groups than in the CON group (*p* < 0.05). Sperm motility in the EP5 group was significantly higher than that in the CON group (*p* < 0.05), while sperm volume tended to increase.

### 3.2. Effect of Dietary Epimedium on Serum Biochemical Indicators of Bama Boars

As presented in Table 3, compared with the CON group, both the EP3 and EP5 treatments exhibited significantly lower serum TBA levels (*p* < 0.01) and significantly higher serum TP levels (*p* < 0.05). Specifically, the EP5 treatment significantly lowered serum ALT levels (*p* < 0.01), while the EP3 treatment significantly raised serum ALB levels (*p* < 0.01) compared to the CON group.

### 3.3. Effect of Dietary Epimedium on Serum Reproductive Hormones of Bama Boars

As shown in Table 4, serum FSH levels were significantly elevated in both the EP3 and EP5 groups compared with the CON group (*p* < 0.01). Notably, the EP5 group also showed significant increases in serum T and LH levels (*p* < 0.05).

### 3.4. Effect of Dietary Epimedium on Antioxidant Capacity of Bama Boars

As presented in Table 5, both the EP3 and EP5 groups exhibited elevated serum SOD activity compared with the CON group (*p* < 0.01), with the EP3 group showing the highest enhancement. Additionally, serum GSH-Px activity was significantly higher in the EP3 group than in the CON group (*p* < 0.05).

### 3.5. Effect of Dietary Epimedium on the Immune Level of Bama Boars

As presented in Table 6, compared with the CON group, both the EP3 and EP5 groups exhibited significantly reduced serum levels of TNF-α and IL-6 (*p* < 0.05). In contrast, the EP5 group showed a significant elevation in serum IgG levels (*p* < 0.05). No significant differences were observed in the levels of IFN-γ, IgA, or IgM among the three groups (*p* > 0.05).

### 3.6. Effects of Dietary Epimedium on the Diversity and Composition of Gut Microbiota in Bama Boars

**Figure 1:**
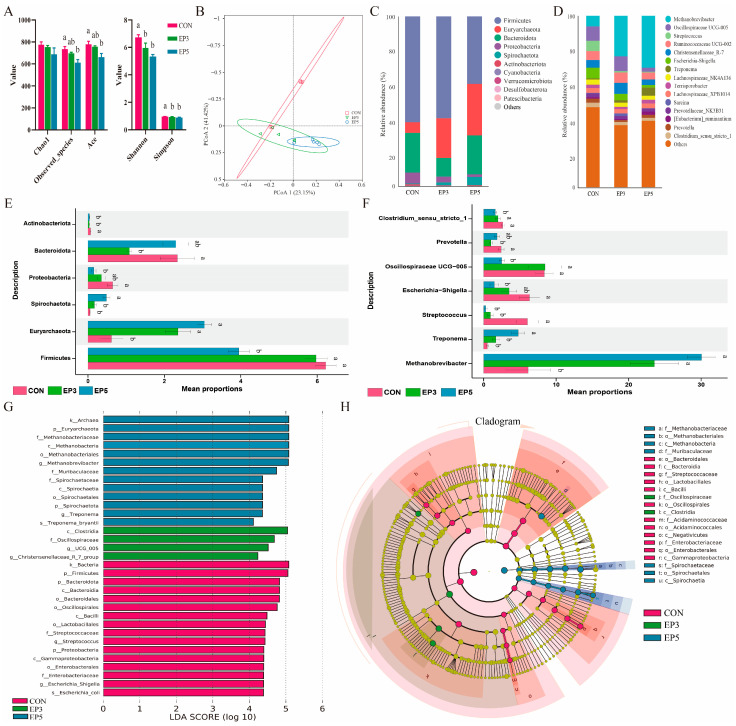
Impact of dietary *Epimedium* on gut microbial diversity and composition in Bama boars. (**A**) Alpha diversity at the OTU level. (**B**) Beta diversity assessed by principal coordinate analysis (PCoA). (**C**,**D**) Microbial taxonomic profiles at the phylum and genus levels, respectively. (**E**,**F**) Analysis of significant differences in microbiota composition at the phylum and genus levels. (**G**,**H**) Linear discriminant analysis effect size (LEfSe) results (LDA score > 4) and branching diagram of evolution scatterplot. Values represent mean ± SD (*n* = 5); distinct superscripts denote significance (*p* < 0.05).

Figure 1A illustrates the α-diversity of the intestinal microbiota in Bama boars across the CON, EP3, and EP5 groups. Notably, the Simpson and Shannon diversity indices decreased in the EP3 and EP5 groups relative to the CON group (*p* < 0.05). Figure 1B depicts the β-diversity analysis, revealing distinct clustering patterns between the treatment groups (EP3 and EP5) and the control. Principal coordinate analysis (PCoA) revealed an overlap between the CON and EP3 groups, whereas both were distinctly separated from the EP5 group. At the phylum level, the three most abundant taxa were *Firmicutes*, *Euryarchaeota*, and *Bacteroidota*. Relative to the CON group, the EP3 and EP5 groups showed significantly elevated *Euryarchaeota* levels but reduced *Actinobacteriota* (*p* < 0.05). Specifically, the EP5 group exhibited increased *Spirochaeta* and decreased *Firmicutes* and *Proteobacteria* (*p* < 0.05), while the EP3 group showed reduced *Bacteroidota* (*p* < 0.05). The most abundant genera identified at the genus level included *Methanobrevibacter*, *Streptococcus*, and *Oscillospiraceae_UCG-005*. Both treatment groups significantly enriched *Methanobrevibacter* and depleted *Streptococcus* relative to the control (*p* < 0.05). Notably, the EP5 group also increased *Treponema* abundance while decreasing *Escherichia-Shigella*, *Oscillospiraceae_UCG-005*, and *Clostridium_sensu_stricto_1* (*p* < 0.05). In contrast, the EP3 group specifically reduced *Prevotella* (*p* < 0.05). LEfSe analysis identified distinct biomarkers for each group. The CON group was characterized by higher LDA scores for the kingdom *Archaea*, phylum *Euryarchaeota*, and family *Methanobacteriaceae*. The EP3 group was enriched in the class *Clostridia*, family *Oscillospiraceae*, genus *Oscillospiraceae_UCG-005*, and genus *Christensenellaceae_R-7_group*. Conversely, the EP5 group showed higher LDA scores for the kingdom *Bacteria*, phylum *Firmicutes*, and phylum *Bacteroidota*.

### 3.7. PICRUSt2 Functional Annotation of Gut Microbiota

**Figure 2:**
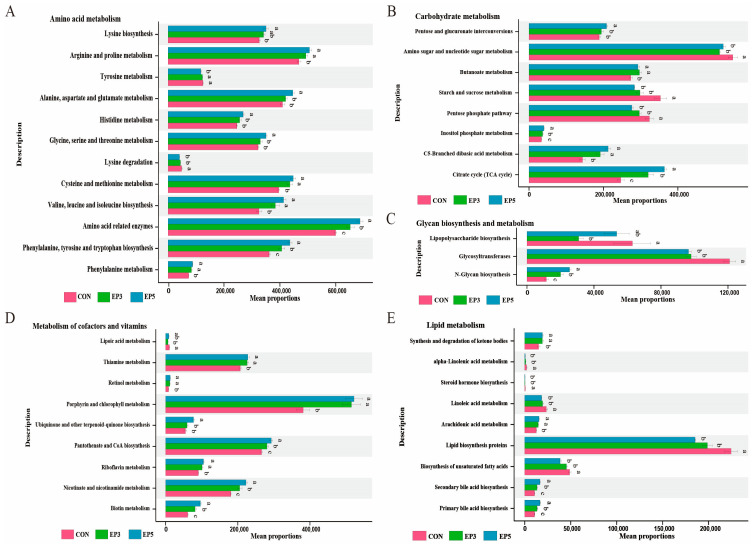
Changes in Bama boars’ predicted function with *Epimedium* supplementation. (**A**–**E**) Differential KEGG functions at pathway level 3 within “Metabolism” among three groups. Values represent mean ± SD (*n* = 5); distinct superscripts denote significance (*p* < 0.05).

Predictive functional analysis was performed using PICRUSt based on KEGG Level 3 pathways. As shown in Figure 2, a total of 41 differential metabolic pathways were identified across 10 major metabolic categories. The result shows 12 “amino acid metabolism”, 8 “carbohydrate metabolism”, 3 “glycan biosynthesis and metabolism”, 9 “metabolism of cofactors and vitamins”, and 9 “lipid metabolism” pathways were significantly different (*p* < 0.05) among the three groups. In amino acid metabolism, compared with the CON group, the EP3 group and EP5 group were mainly enriched in the pathways of phenylalanine metabolism, phenylalanine, tyrosine, and tryptophan biosynthesis; amino acid-related enzymes; valine, leucine, and isoleucine biosynthesis; cysteine and methionine metabolism; and arginine and proline metabolism, while down-regulating the lysine degradation pathway (*p* < 0.05). In carbohydrate metabolism, compared with the CON group, the EP3 group and EP5 group were mainly enriched in the citrate cycle (TCA cycle), C5-branched dibasic acid metabolism, inositol phosphate metabolism, and butanoate metabolism pathways but were downregulated in the pentose phosphate pathway and starch and sucrose metabolism pathways (*p* < 0.05). Regarding cofactor and vitamin metabolism, the EP3 and EP5 groups exhibited enrichment in the pathways of biotin metabolism, nicotinate and nicotinamide metabolism, riboflavin metabolism, pantothenate and CoA biosynthesis, porphyrin and chlorophyll metabolism, retinol metabolism, and thiamine metabolism (*p* < 0.05). In glycan biosynthesis and metabolism, the EP3 group and the EP5 group were enriched in the N-glycan biosynthesis pathway while being downregulated in the glycosyltransferases pathway (*p* < 0.05). In lipid metabolism, compared with the CON group, the EP3 group and the EP5 group showed upregulation in the primary bile acid biosynthesis, secondary bile acid biosynthesis, arachidonic acid metabolism, and synthesis and degradation of ketone bodies pathways but downregulation in the biosynthesis of lipid biosynthesis proteins, linoleic acid metabolism, and steroid hormone biosynthesis pathways (*p* < 0.05).

### 3.8. Fecal Metabolite Profiles in Bama Boars

**Figure 3:**
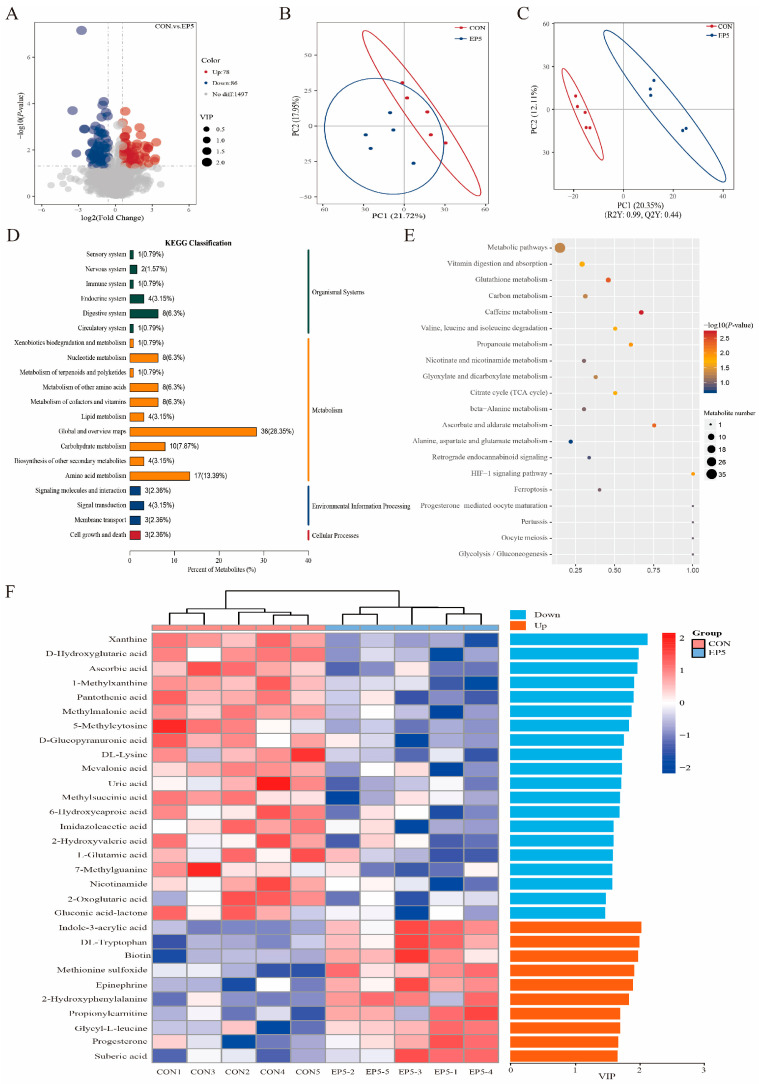
Metabolomic profiling and classification of the CON and EP5 groups. (**A**) Volcano plot of differential metabolites; (**B**) Scatter plot of PCA scores; (**C**) Partial least-squares discriminant analysis (PLS-DA) score plot; (**D**) KEGG classification of differentially expressed metabolites; (**E**) Metabolome view map of common metabolites identified in the two groups; (**F**) Hierarchical cluster analysis heat map of significant differential metabolites.

The differences in metabolites between the CON group and the EP5 group were analyzed using non-targeted metabolomics methods. The volcano diagram in Figure 3A shows the 164 differentially expressed metabolites between the CON and EP5 groups, including 78 upregulated metabolites and 86 downregulated metabolites. The PCA analysis and PLS-DA showed that the microbial community structures of the CON and EP5 groups differed substantially. The PLS-DA models were authenticated by cross-validation among CON vs EP5 (R^2^  =  0.99 and Q^2^  =  0.44). KEGG functional classification revealed that the CON and EP5 groups were primarily enriched in ‘global and overview maps’, ‘amino acid metabolism’, and ‘carbohydrate metabolism’ (Figure 3D). KEGG enrichment analysis demonstrated that the most prominently affected metabolic pathways were related to vitamin digestion and absorption, glutathione metabolism, valine, leucine, and isoleucine degradation, propanoate metabolism, and so on. Metabolites were considered significantly influenced by *Epimedium* supplementation if they met the criteria of VIP > 1 and *p* < 0.05 (Figure 3F). The heatmap of the differential metabolites shows that mainly 20 differentially expressed metabolites, including xanthine, D-α-hydroxyglutaric acid, methylmalonic acid, and uric acid, showed significant downregulation, whereas 10 differentially expressed metabolites, including indole-3-acrylic acid, DL-tryptophan, 2-hydroxyphenylalanine, and propionylcarnitine, showed significant upregulation after *Epimedium* supplementation.

### 3.9. Spearman Correlation Among Fecal Microbes and Metabolites, Serum Parameters, and Semen Quality

**Figure 4:**
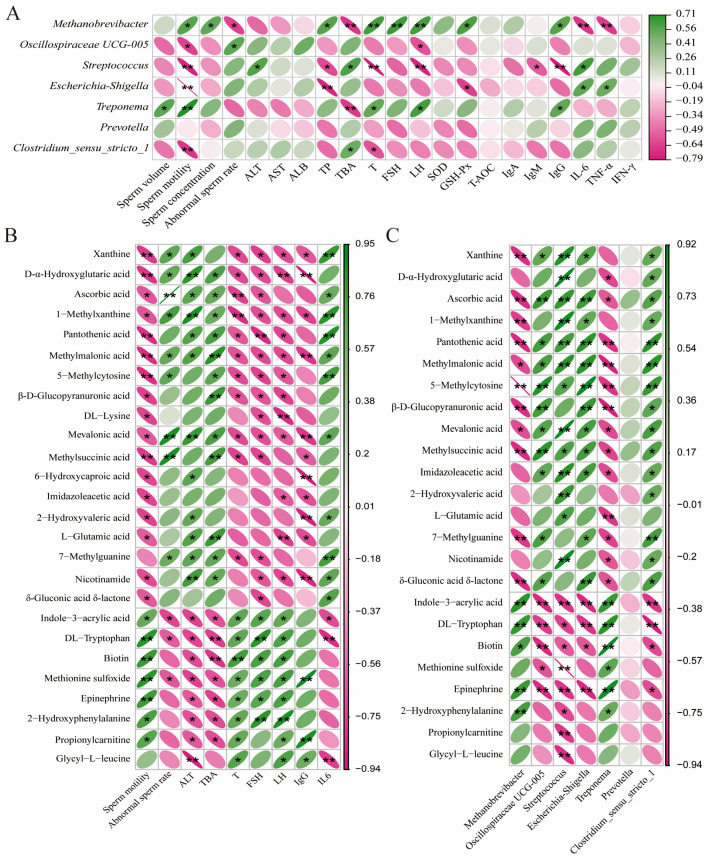
Spearman correlation analysis among fecal microbiota, fecal metabolites, semen quality, and serum parameters. (**A**) Associations between dominant differential microbial taxa and semen/serum parameters. (**B**) Correlations of dominant differential metabolites with semen quality and serum parameters. (**C**) Interactions between dominant differential metabolites and gut microbial taxa. Asterisks indicate statistical significance: * for *p* < 0.05 and ** for *p* < 0.01 (*n* = 5).

Spearman correlation analysis (Figure 4) revealed significant associations among fecal microbiota, metabolites, serum biochemical parameters, and semen quality indices. According to Figure 4A, there was a negative correlation between the relative abundance of *Methanobrevibacter* and abnormal sperm rate, TBA, IL-6, and TNF-α, and a positive correlation with semen quality (sperm motility and sperm concentration) and serum-related parameters (TP, T, FSH, LH, GSH-Px, and IgG). The relative abundance of *Oscillospiraceae UCG-005* shows a negative correlation with sperm motility and LH and a positive correlation with the abnormal sperm rate. The relative abundances of *Streptococcus* and *Clostridium_sensu_stricto_1* were negatively correlated with sperm motility and serum T, but positively correlated with TBA. Furthermore, Streptococcus showed a positive association with ALT and IL-6, while exhibiting negative correlations with beneficial serum parameters, including TP, LH, IgM, and IgG. *Escherichia-Shigella*’s relative abundance had a positive correlation with IL-6 and TNF-α and a negative correlation with sperm motility, TP, and GSH-Px. The relative abundance of *Treponema* shows a negative correlation with TBA and a positive correlation with sperm volume, sperm motility, T, LH, and IgG. Next, there is a strong correlation between these fecal metabolites and serum parameters as well as semen quality (Figure 4B). Sperm motility and reproductive hormones (T, FSH, and LH) show a positive correlation with indole-3-acrylic acid, DL-tryptophan, biotin, methionine sulfoxide, epinephrine, and 2-hydroxyphenylalanine, while they show a negative correlation with xanthine, D-α-hydroxyglutaric acid, 1-methylxanthine, pantothenic acid, methylmalonic acid, 5-methylcytosine, β-D-glucopyranuronic acid, and mevalonic acid. The abnormal sperm rate and serum biochemical indicators (ALT and TBA) showed a negative correlation with indole-3-acrylic acid, DL-tryptophan, and methionine sulfoxide, while they showed a positive correlation with D-α-hydroxyglutaric acid, ascorbic acid, 1-methylxanthine, methylmalonic acid, mevalonic acid, and 7-methylguanine. The content of serum IgG shows a positive correlation with glycyl-L-leucine and a negative correlation with xanthine, 1-methylxanthine, methylmalonic acid, mevalonic acid, 2-hydroxyvaleric acid, and nicotinamide. However, the content of serum IL-6 shows an opposite correlation with these metabolites. Furthermore, there is also a strong correlation between the fecal metabolites and gut microbes (Figure 4C). The relative abundances of Methanobrevibacter and Treponema were positively correlated with indole-3-acrylic acid, DL-tryptophan, biotin, epinephrine, and 2-hydroxyphenylalanine, while negatively correlated with ascorbic acid, pantothenic acid, methylmalonic acid, 5-methylcytosine, β-D-glucopyranuronic acid, mevalonic acid, methylsuccinic acid, 7-methylguanine, and δ-gluconic acid δ-lactone. The relative abundances of *Oscillospiraceae UCG-005*, *Streptococcus*, *Escherichia-Shigella*, and *Clostridium_sensu_stricto_1* were positively correlated with xanthine, ascorbic acid, pantothenic acid, methylmalonic acid, 5-methylcytosine, mevalonic acid, methylsuccinic acid, and imidazoleacetic acid, while negatively correlated with indole-3-acrylic acid, DL-tryptophan, biotin, and epinephrine.

## 4. Discussion

In the swine industry, semen quality serves as a critical determinant of breeding efficiency. Traditional Chinese medicines (TCMs) have demonstrated remarkable efficacy in ameliorating male infertility. Notably, *Epimedium* has been extensively employed to enhance male reproductive performance. Its beneficial effects include regulating reproductive endocrine function, promoting sexual libido, restoring spermatogenic function, improving sperm quality, modulating the hypothalamic–pituitary–testicular (HPT) axis, and enhancing erectile function [22,23]. In this experiment, adding 0.3% and 0.5% *Epimedium* to the diet reduced the sperm abnormality rate of the Bama boars. Particularly, the 0.5% dose of *Epimedium* significantly increased sperm motility and showed a tendency to increase sperm volume. As the major flavonoid glycoside of *Epimedium*, icariin (ICA) significantly improves testicular function and spermatogenesis in rodents and goats [24]. Moreover, a 35-day oral regimen of ICA in normal adult rats resulted in significant improvements in epididymal sperm counts, testosterone production, and Sertoli cell function [25]. According to research, the administration of icariin enhanced spermatogenesis and sperm motility and improved testicular organ coefficients [12]. This indicates that Epimedium improves the semen quality of Bama boars, and this is mainly attributed to the active components of *Epimedium*, such as flavonoids, polyphenols, and so on.

Hormones are pivotal environmental factors for germ cell development, synergistically supporting both testicular metabolic homeostasis and spermatogenesis [26]. Serum sex hormones play a pivotal role in regulating physiological functions. For instance, FSH and LH stimulate gonadal development and gametogenesis, whereas T is a steroid hormone primarily synthesized by the testes and adrenal glands [27]. In this experiment, the addition of 0.3% and 0.5% of *Epimedium* increased the level of serum FSH, and 0.5% of Epimedium also raised the levels of T and LH in the serum. Studies have demonstrated that administering 50 mg/kg of icariin significantly enhanced the reproductive performance of male dairy goats, mediated by elevated ss levels of GnRH, LH, and T [12]. Icariin is known to exert androgenic effects, significantly increasing circulating testosterone concentrations in rats [28]. Thus, our results are similar to those of the previous studies, indicating that *Epimedium* may regulate the reproductive functions of Bama boars by increasing the levels of reproductive hormones.

A large number of research reports have stated that flavonoids have a protective effect on male reproductive system dysfunction [29]. *Epimedium* is rich in flavonoids, such as icariin. Flavonoids have been reported to have significant antioxidant, anti-inflammatory, and immunostimulatory effects [10]. Based on this, we further investigated the effects of adding *Epimedium* to the diet on the antioxidant and immune functions of Bama boars. The endogenous antioxidant defense system relies on key enzymes like SOD, GPx, and GST. These enzymes function synergistically to scavenge reactive oxygen species (ROS) and counteract oxidative stressors, thus preserving cellular integrity from oxidative damage [30]. In this experiment, adding 0.3% and 0.5% of *Epimedium* to the diet increased the activity of SOD in the serum, while 0.3% of *Epimedium* increased the activity of GSH-Px in the serum. Accumulating evidence demonstrates that flavonoid supplementation upregulates endogenous antioxidant defense systems, particularly through activation of SOD, CAT, and GSH-Px enzymatic pathways [31]. In rat models, icariin from *Epimedium* reduces oxidative stress by upregulating SOD, CAT, GPx, and glutathione reductase [32]. Similarly, icariin supplementation in goats has been shown to significantly increase serum levels of key enzymes, including SOD, GPx, and CAT [12]. Serum immunoglobulins, including IgA, IgG, and IgM, are vital biomarkers of immune status [33]. IL-6 and TNF-α serve as key pro-inflammatory mediators released primarily by activated monocytes and macrophages. These cytokines stimulate the secretion of other inflammatory mediators, amplifying the inflammatory response [34]. In this experiment, adding 0.3% and 0.5% of *Epimedium* to the diet reduced the levels of serum TNF-α and IL-6. Interestingly, adding 0.5% of *Epimedium* actually increased the level of IgG in the serum. Zhang et al. [17] reported that supplementing the diet with *Epimedium* (200 mg/kg) elevated serum immunoglobulin (IgA and IgG) levels in broilers. Previous studies have reported that icariin administration markedly reduced pro-inflammatory mediators (IL-1β, IL-6, and TNF-α) and elevated IL-10 levels [35,36]. Overall, our results are similar to those reported above, indicating that *Epimedium* may regulate the reproductive functions of Bama boars through its antioxidant and immune properties.

Emerging evidence has increasingly highlighted the critical influence of the gut microbiota on spermatogenesis and semen quality [15]. Intervention with *Epimedium* led to a reduction in the Shannon and Simpson indices, reflecting a decrease in intestinal microbial α-diversity. This might be related to the individual differences among boars, and further investigation is needed. In this experiment, adding 0.5% of *Epimedium* to the diet resulted in a decrease in the abundance of *Proteobacteria* at the phylum level. Moreover, adding 0.5% of *Epimedium* to the diet decreased the abundance of *Bacteroidota*. Research indicates that *Epimedium* reshaped the gut microbial community in Chinese forest musk deer by boosting alpha diversity and suppressing the proliferation of potentially pathogenic *Proteobacteria* [37]. Another study has reported that increased relative abundances of *Bacteroides* and *Prevotella* were positively associated with elevated circulating endotoxin levels and impaired spermatogenesis [15]. At the genus level in this experiment, it was observed that adding 0.3% of *Epimedium* reduced the relative abundance of *Prevotella*. Adding *Epimedium* to the diet significantly reduced the relative abundance of *Streptococcus* in the intestines of Bama boars. Among them, 0.5% *Epimedium* reduced the relative abundance of *Escherichia-Shigella*, *Oscillospiraceae UCG-005*, and *Clostridium_sensu_stricto_1*. Previous studies have reported that adding a polyphenol (hydroxytyrosol) to the diet of boars reduced the relative abundance of “harmful microorganisms” such as *Streptococcus*, *Oscillibacter*, *Clostridium_sensu_stricto*, and *Escherichia* [38]. Overall, our data results indicated that *Epimedium* might have the potential to enhance spermatogenesis by benefiting the gut microbiota of Bama boars.

Studies have demonstrated that host–gut microbiota interactions modulate semen quality through the production of key metabolites, including SCFAs (e.g., butyrate), amino acids, vitamins, and bile acids [39]. PICRUSt2 analysis revealed that the intestinal microbiota of boars in the Epimedium group was primarily enriched in metabolic pathways, including the biosynthesis of phenylalanine, tyrosine, and tryptophan, the synthesis of valine, leucine, and isoleucine, inositol phosphate metabolism, butyric acid metabolism, biotin metabolism, and arachidonic acid metabolism. Studies have demonstrated that butyric acid and its salts (butyrate) possess potent immunomodulatory properties, contribute to intestinal homeostasis, and serve as a critical carbon source for intestinal epithelial cells [40]. A study underscored the pivotal role of amino acid metabolic pathways in regulating key indices of semen quality [41]. Previous studies have reported that the levels of several amino acids, including leucine (Leu), glutamic acid (Glu), and tryptophan (Trp), are significantly reduced in patients with asthenozoospermia [42]. Studies have shown that individual amino acids may play a critical role in modulating semen quality [43]. Consistent with previous findings, *Epimedium* supplementation enhanced boar health by modulating lipid, energy, and amino acid metabolism, thereby increasing the levels of beneficial metabolites [44]. In the current investigation, LC-MS/MS-based nontargeted metabolomics analysis revealed that dietary supplementation of *Epimedium* significantly upregulated the levels of differential metabolites related to the indole-3-acrylic acid, DL-tryptophan, 2-hydroxyphenylalanine, and propionylcarnitine. As a precursor of indole-3-propionic acid (IPA), the enhancement of tryptophan metabolism further improves the bioavailability of IPA [45]. IPA has anti-inflammatory, antioxidant, and immunomodulatory properties [46]. In the current study, it was observed that sperm motility and reproductive hormones (T, FSH, and LH) were positively correlated with indole-3-acrylic acid, DL-tryptophan, biotin, and 2-hydroxyphenylalanine. Indole-derived metabolites upregulate the expression of CatSper protein and enhance testosterone secretion, thereby alleviating testicular damage caused by cisplatin (II), inhibiting oxidative stress (OS) and inflammation, and restoring hormone levels [47]. In a prior study, DL-tryptophan, an essential amino acid supplement, was shown to improve intestinal morphology and enhance the intestinal immune system [48]. Furthermore, tryptophan significantly improves ram sperm motility [49]. Supplementation with antioxidants such as carotenoids and L-carnitine can modulate gut microbiota, reducing sperm damage induced by oxidative stress [50]. In this study, it was also found that the relative abundances of *Oscillospiraceae UCG-005*, *Streptococcus*, *Escherichia-Shigella*, and *Clostridium_sensu_stricto_1* were negatively correlated with indole-3-acrylic acid, DL-tryptophan, biotin, and epinephrine. Conversely, the robust positive correlations between these beneficial microbial taxa and semen quality parameters (motility and density) imply that the gut microbiota serves as a pivotal mediator linking dietary intervention to enhanced reproductive performance [30]. The flavonoids and diverse bioactive constituents of Epimedium exert beneficial effects on digestive efficiency and immune function via the modulation of gut microbial community structure and metabolic activity [51]. In the current study, the relative abundances of *Streptococcus* and *Clostridium_sensu_stricto_1* were negatively correlated with sperm motility and serum testosterone (T). Furthermore, the relative abundance of *Escherichia-Shigella* showed a positive correlation with pro-inflammatory cytokines (IL-6 and TNF-α) and a negative correlation with sperm motility. Emerging evidence suggests that microbially derived metabolites, such as SCFAs, in conjunction with immune–regulatory pathways, play a pivotal role in modulating energy metabolism and reproductive function [52]. Taken together, these data suggest that *Epimedium* has the potential to optimize the intestinal metabolome, thereby benefiting spermatogenesis. Further research is needed to elucidate the underlying regulatory mechanisms and systemic interactions governing these effects.

The sample size of this study was limited (*n* = 6 boars per group), which may reduce the statistical power and limit the generalizability of the findings regarding fecal microbiota, metabolites, serum parameters, and semen quality. Individual variability among the boars further exacerbated these limitations. Therefore, future studies should prioritize validating these results in larger cohorts and extending the *Epimedium* intervention duration to confirm the stability and broader applicability of our conclusions. Furthermore, further research is needed to elucidate the mechanisms by which *Epimedium* mediates the ‘gut–testis’ axis, specifically its regulation of the testicular microenvironment and spermatogenesis.

## 5. Conclusions

Dietary *Epimedium*, especially 0.5%, improved selected semen, hormone, immune, antioxidant, microbiota, and fecal metabolite indices in Bama boars, while further studies are needed to validate fertility outcomes and mechanisms.

## Figures and Tables

**Table 1:** The ingredient and nutrient composition of the basal diet (% as fed basis).

Ingredients	Content (%)
Corn	52.88
Soybean meal	13.39
Rice bran	8.57
Whey	8.22
Soybean oil	3.0
Sugar	8
Limestone	1.4
Attapulgite	0.96
Salt	2.20
*L*-Lysine	0.20
*L*-Threonine	0.71
Premixes ^1^	0.47
Total	100
Nutrient composition ^2^	
Calculated NE, kcal/kg	3.36
Crude Fiber	4.15
Crude protein	13.00
Calcium	0.79
Phosphorus	0.60
Lysine	1.00
Threonine	1.20
Isoleucine	0.49
Methionine & Cysteine	0.43

^1^ The premix per kilogram of feed contains vitamin A 4000 IU, vitamin D3 400 IU, vitamin E 18 mg, vitamin K3 0.5 mg, vitamin B2 1.5 mg, vitamin B12 8 ug, pantothenic acid 1.8 mg, niacin 1.8 mg, Fe 200 mg, Cu 20 mg, Mn 20 mg, Zn 20 mg, I 1 mg/kg, Se 0.2 mg. ^2^ Nutrient levels were calculated.

**Table 2:** Effect of *Epimedium* on semen quality in Bama boars.

Items	CON	EP3	EP5	*p*-Value
Sperm volume, mL	168.80 ± 21.23	172.60 ± 25.80	207.20 ± 28.63	0.065
Sperm motility, %	85.17 ± 4.34 ^b^	89.40 ± 2.64 ^ab^	91.76 ± 2.26 ^a^	0.021
Sperm concentration, 10^8^ sperm/mL	1.90 ± 1.01	3.06 ± 1.37	3.74 ± 2.98	0.363
Abnormal sperm rate, %	3.38 ± 1.57 ^a^	1.56 ± 1.31 ^b^	1.05 ± 0.68 ^b^	0.029

Different superscript letters differ significantly (*p* < 0.05; *n* = 6).

**Table 3:** Effect of *Epimedium* on serum biochemical indicators in Bama boars.

Items	CON	EP3	EP5	*p*-Value
AST (U/L)	52.08 ± 8.80	45.26 ± 10.02	41.58 ± 13.18	0.332
ALT (U/L)	38.98 ± 2.93 ^a^	35.72 ± 5.49 ^a^	26.80 ± 5.71 ^b^	0.005
TBA (μmol/L)	6.14 ± 1.76 ^a^	3.30 ± 0.96 ^b^	2.78 ± 0.98 ^b^	0.009
TP (g/L)	72.45 ± 3.95 ^b^	81.10 ± 4.77 ^a^	78.94 ± 4.49 ^a^	0.024
ALB (g/L)	52.72 ± 1.75 ^b^	58.70 ± 5.06 ^a^	50.42 ± 3.08 ^b^	0.003

Different superscript letters differ significantly (*p* < 0.05; *n* = 6).

**Table 4:** Effect of *Epimedium* on serum hormone indicators in Bama boars.

Items	CON	EP3	EP5	*p*-Value
T (pg/mL)	207.55 ± 28.21 ^b^	247.71 ± 46.54 ^ab^	293.84 ± 62.83 ^a^	0.024
LH (mIU/mL)	8.51 ± 2.32 ^b^	10.70 ± 2.83 ^b^	14.58 ± 3.07 ^a^	0.006
FSH (mIU/mL)	10.88 ± 2.41 ^b^	16.24 ± 3.87 ^a^	15.52 ± 2.77 ^a^	0.017

Different superscript letters differ significantly (*p* < 0.05; *n* = 6).

**Table 5:** Effect of Epimedium on serum antioxidant indicators in Bama boars.

Items	CON	EP3	EP5	*p*-Value
GSH-Px (U/mL)	56.75 ± 15.31 ^b^	90.65 ± 25.15 ^a^	74.07 ± 13.49 ^ab^	0.044
SOD (U/mL)	11.90 ± 4.22 ^b^	19.52 ± 2.86 ^a^	16.81 ± 2.28 ^a^	0.003
T-AOC (U/mL)	0.18 ± 0.01	0.22 ± 0.06	0.21 ± 0.08	0.544

Different superscript letters differ significantly (*p* < 0.05; *n* = 6).

**Table 6:** Effect of *Epimedium* on serum immunoglobulins and cytokines in Bama boars.

Items	CON	EP3	EP5	*p*-Value
IgA (μg/mL)	6.98 ± 1.84	8.05 ± 1.50	9.28 ± 2.22	0.196
IgG (g/L)	3.97 ± 1.73 ^b^	6.69 ± 1.16 ^ab^	9.97 ± 4.81 ^a^	0.027
IgM (μg/mL)	8.99 ± 2.03	10.34 ± 0.99	11.42 ± 3.37	0.295
TNF-α (pg/mL)	86.04 ± 20.21 ^a^	56.20 ± 9.10 ^b^	64.77 ± 14.18 ^b^	0.025
IFN-γ (pg/mL)	41.71 ± 8.39	40.08 ± 12.47	34.89 ± 13.00	0.629
IL-6 (pg/mL)	69.19 ± 19.76 ^a^	25.31 ± 4.71 ^b^	29.97 ± 9.68 ^b^	0.041

Different superscript letters differ significantly (*p* < 0.05; *n* = 6).

## Data Availability

The names of the repository/repositories and accession numbers can be found below: https://www.ncbi.nlm.nih.gov/sra/PRJNA1433486.

## References

[1] Singh M., Talimoa Mollier R., Sharma P.R., Kadirvel G., Doley S., Sanjukta R.K., Rajkhowa D.J., Kandpal B.K., Kumar D., Khan M.H. (2021). Dietary flaxseed oil improves boar semen quality, antioxidant status and in-vivo fertility in humid sub-tropical region of North East India. Theriogenology.

[2] Kholif A.E., Hassan A.A., El Ashry G.M., Bakr M.H., El-Zaiat H.M., Olafadehan O.A., Matloup O.H., Sallam S.M.A. (2021). Phytogenic feed additives mixture enhances the lactational performance, feed utilization and ruminal fermentation of Friesian cows. Anim. Biotechnol..

[3] Ganjalikhan Hakemi S., Sharififar F., Haghpanah T., Babaee A., Eftekhar-Vaghefi S.H. (2019). The effects of olive leaf extract on the testis, sperm quality and testicular germ cell apoptosis in male rats exposed to busulfan. Int. J. Fertil. Steril..

[4] Petrova M., Yordanova G., Eneva K., Nedeva R., Nedelkov K., Penev T. (2026). The Effect of *Turnera diffusa* Leaf Supplementation in Diet on the Qualitative and Quantitative Characteristics of Boar Semen. Life.

[5] Li Y., Wang H., Hu Z., Zhang G., Wen F., Xian M., Guo S., Zhang G., Zhang X., Hu J. (2025). Supplementation of Epimedium polysaccharide (EPS) improves goat semen characteristics following cryopreservation. Anim. Reprod. Sci..

[6] Yang Q., Xing Y., Qiao C., Liu W., Jiang H., Fu Q., Zhou Y., Yang B., Zhang Z., Chen R. (2019). Semen quality improvement in boars fed with supplemental wolfberry (*Lycium barbarum*). Anim. Sci. J..

[7] Zhang J., Zhang Y., Gong H., Cui L., Huang T., Ai H., Ren J., Huang L., Yang B. (2017). Genetic mapping using 1.4M SNP array refined loci for fatty acid composition traits in Chinese Erhualian and Bamaxiang pigs. J. Anim. Breed. Genet..

[8] Huang Y., Zhou L., Zhang J., Liu X., Zhang Y., Cai L., Zhang W., Cui L., Yang J., Ji J. (2020). A large-scale comparison of meat quality and intramuscular fatty acid composition among three Chinese indigenous pig breeds. Meat Sci..

[9] Sze S.C.W., Tong Y., Ng T.B., Cheng C.L.Y., Cheung H.P. (2010). *Herba Epimedii*: Anti-oxidative properties and its medical implications. Molecules.

[10] Szabo R., Racz C.P., Dulf F.V. (2022). Bioavailability improvement strategies for icariin and its derivatives: A review. Int. J. Mol. Sci..

[11] Park H., Koo Y., Park M., Hwang Y., Hwang S., Park N.C. (2017). Restoration of spermatogenesis using a new combined herbal formula of *Epimedium koreanum* nakai and *Angelica gigas* nakai in an luteinizing Hormone-Releasing hormone Agonist-Induced rat model of male infertility. World J. Men’s Health.

[12] Zhao F., Chen H., Wang S., Zhang X., Chen N., Chen H., Fu J., Liu H., Liu J., Liu T. (2024). Effects of icariin as a feed additive on the reproductive function in bucks (*Capra hircus*). Front. Vet. Sci..

[13] Wang Y., Xie Z. (2022). Exploring the role of gut microbiome in male reproduction. Andrology.

[14] Chen W., Zou H., Xu H., Cao R., Zhang H., Zhang Y., Zhao J. (2024). The potential influence and intervention measures of gut microbiota on sperm: It is time to focus on the testis-gut microbiota axis. Front. Microbiol..

[15] Ding N., Zhang X., Zhang X.D., Jing J., Liu S.S., Mu Y.P., Peng L.L., Yan Y.J., Xiao G.M., Bi X.Y. (2020). Impairment of spermatogenesis and sperm motility by the high-fat diet-induced dysbiosis of gut microbes. Gut.

[16] Guo Q., Cheng Y., Li T., Huang J., Li J., Zhang Z., Qu Y. (2024). The gut microbiota contributes to the development of LPS-Induced orchitis by disrupting the Blood-Testosterone barrier in mice. Reprod. Sci..

[17] Zhang J., Yu H., Zhang H., Zhao Q., Si W., Qin Y., Zhang J. (2023). Dietary Epimedium extract supplementation improves intestinal functions and alters gut microbiota in broilers. J. Anim. Sci. Biotechnol..

[18] Guo L., Wu Y., Wang C., Wei H., Tan J., Sun H., Jiang S., Peng J. (2020). Gut microbiological disorders reduce semen utilization rate in duroc boars. Front. Microbiol..

[19] Liu M., Chen R., Wang T., Ding Y., Zhang Y., Huang G., Huang J., Qu Q., Lv W., Guo S. (2024). Dietary Chinese herbal mixture supplementation improves production performance by regulating reproductive hormones, antioxidant capacity, immunity, and intestinal health of broiler breeders. Poult. Sci..

[20] Liu M., Zhou J., Li Y., Ding Y., Lian J., Dong Q., Qu Q., Lv W., Guo S. (2023). Effects of dietary polyherbal mixtures on growth performance, antioxidant capacity, immune function and jejunal health of yellow-feathered broilers. Poult. Sci..

[21] Liu J., Stewart S.N., Robinson K., Yang Q., Lyu W., Whitmore M.A., Zhang G. (2021). Linkage between the intestinal microbiota and residual feed intake in broiler chickens. J. Anim. Sci. Biotechnol..

[22] Zhang W., Chen H., Wang Z., Lan G., Zhang L. (2013). Comparative studies on antioxidant activities of extracts and fractions from the leaves and stem of *Epimedium koreanum* Nakai. J. Food Sci. Technol..

[23] Zhao H., Zhao T., Yang J., Huang Q., Wu H., Pan Y., Wang H., Qian Y. (2022). Epimedium protects against dyszoospermia in mice with Pex3 knockout by exerting antioxidant effects and regulating the expression level of P16. Cell Death Dis..

[24] Zhang J., Zhang C., Liu A., Ji Q., Ren L., Ma C., Zhang H., Wu C., Zhang D., Shang M. (2021). Synthesis of Icariin-Zinc and its protective effect on exercise fatigue and reproductive system-related glands in male rats. Front. Pharmacol..

[25] Chen M., Hao J., Yang Q., Li G. (2014). Effects of icariin on reproductive functions in male rats. Molecules.

[26] Ruwanpura S.M., McLachlan R.I., Meachem S.J. (2010). Hormonal regulation of male germ cell development. J. Endocrinol..

[27] Lei T., Yang Y., Yang W. (2025). Luteinizing hormone regulates testosterone production, Leydig cell proliferation, differentiation, and circadian rhythm during spermatogenesis. Int. J. Mol. Sci..

[28] Zhang Z., Yang Q. (2006). The testosterone mimetic properties of icariin. Asian J. Androl..

[29] Ye R., Yang J., Hai D., Liu N., Ma L., Lan X., Niu J., Zheng P., Yu J. (2020). Interplay between male reproductive system dysfunction and the therapeutic effect of flavonoids. Fitoterapia.

[30] Zhang T., Sun P., Geng Q., Fan H., Gong Y., Hu Y., Shan L., Sun Y., Shen W., Zhou Y. (2021). Disrupted spermatogenesis in a metabolic syndrome model: The role of vitamin A metabolism in the gut-testis axis. Gut.

[31] Leung W., Yang M., Lee S., Kuo C., Ho Y., Huang-Liu R., Lin H., Kuan Y. (2017). Protective effect of zerumbone reduces lipopolysaccharide-induced acute lung injury via antioxidative enzymes and Nrf2/HO-1 pathway. Int. Immunopharmacol..

[32] El-Shitany N.A., Eid B.G. (2019). Icariin modulates carrageenan-induced acute inflammation through HO-1/Nrf2 and NF-kB signaling pathways. Biomed. Pharmacother..

[33] Wang W., Chen J., Zhou H., Wang L., Ding S., Wang Y., Song D., Li A. (2017). Effects of microencapsulated *Lactobacillus plantarum* and fructooligosaccharide on growth performance, blood immune parameters, and intestinal morphology in weaned piglets. Food Agric. Immunol..

[34] De Martinis M., Franceschi C., Monti D. (2005). Inflamm-aging and lifelong antigenic load as major determinants of aging rate and longevity. FEBS Lett..

[35] Bi Z., Zhang W., Yan X. (2022). Anti-inflammatory and immunoregulatory effects of icariin and icaritin. Biomed. Pharmacother..

[36] Shaukat A., Shaukat I., Rajput S.A., Shukat R., Hanif S., Huang S., Aleem M.T., Li K., Li Q., Chen C. (2022). Icariin alleviates *Escherichia coli* Lipopolysaccharide-Mediated endometritis in mice by inhibiting inflammation and oxidative stress. Int. J. Mol. Sci..

[37] Xie S., Yang Q., Ying Z., Cai M., Fan W., Gao H., Feng X., Wu Y. (2025). Dietary supplementation with Epimedium contributes to the improvement of hormone levels, gut microbiota, and serum metabolite composition in the Chinese forest musk deer (*Moschus berezovskii*). Front. Vet. Sci..

[38] Han H., Zhong R., Zhou Y., Xiong B., Chen L., Jiang Y., Liu L., Sun H., Tan J., Tao F. (2021). Hydroxytyrosol benefits boar semen quality via improving gut microbiota and blood metabolome. Front. Nutr..

[39] Li J., Li Y., Cheng M., Ye F., Li W., Wang C., Huang Y., Wu Y., Xuan R., Liu G. (2022). Gut microbial diversity among Yorkshire, Landrace and Duroc boars and its impact on semen quality. AMB Express.

[40] Zhang J., Zhao Q., Qin Y., Si W., Zhang H., Zhang J. (2023). The effect of epimedium isopentenyl flavonoids on the broiler gut health using microbiomic and metabolomic analyses. Int. J. Mol. Sci..

[41] Boguenet M., Bocca C., Bouet P., Serri O., Chupin S., Tessier L., Blanchet O., El Hachem H., Chao De La Barca J.M., Reynier P. (2020). Metabolomic signature of the seminal plasma in men with severe oligoasthenospermia. Andrology.

[42] Zhao K., Zhang J., Xu Z., Xu Y., Xu A., Chen W., Miao C., Liu S., Wang Z., Jia R. (2018). Metabolomic profiling of human spermatozoa in idiopathic asthenozoospermia patients using gas Chromatography-Mass spectrometry. BioMed Res. Int..

[43] Ren B., Cheng X., Wu D., Xu S., Che L., Fang Z., Lv G., Dong H., Lin Y. (2015). Effect of different amino acid patterns on semen quality of boars fed with low-protein diets. Anim. Reprod. Sci..

[44] Pan S., Chen A., Han Z., Wang Y., Lu X., Yang Y. (2016). 1H NMR-based Metabonomic Study on the Effects of Epimedium on Glucocorticoid-induced Osteoporosis. J. Chromatogr. B.

[45] Xu H., Luo Y., An Y., Wu X. (2025). The mechanism of action of indole-3-propionic acid on bone metabolism. Food Funct..

[46] Li J., Zhang L., Wu T., Li Y., Zhou X., Ruan Z. (2021). Indole-3-propionic acid improved the intestinal barrier by enhancing the epithelial barrier and mucus barrier. J. Agric. Food Chem..

[47] Afsar T., Razak S., Trembley J.H., Khan K., Shabbir M., Almajwal A., Alruwaili N.W., Ijaz M.U. (2022). Prevention of testicular damage by indole derivative MMINA via upregulated StAR and CatSper channels with coincident suppression of oxidative stress and inflammation: In silico and in vivo validation. Antioxidants.

[48] Xie K., Feng X., Zhu S., Liang J., Mo Y., Feng X., Ye S., Zhou Y., Shu G., Wang S. (2024). Effects of tryptophan supplementation in diets with different protein levels on the production performance of broilers. Animals.

[49] Pichardo A., Tlachi-López J., Trejo F., Fuentes A., Báez-Saldaña A., Molina-Cerón M., Manjarrez G., Gutiérrez-Ospina G., Lucio R. (2011). Increased serotonin concentration and tryptophan hydroxylase activity in reproductive organs of copulator males: A case of adaptive plasticity. Adv. Biosci. Biotechnol..

[50] Li X., Meng L., Shen L., Ji H. (2023). Regulation of gut microbiota by vitamin C, vitamin E and beta-carotene. Food Res. Int..

[51] Li B., Xiang T., Bindawa Isah M., Chen C., Zhang X. (2024). In vitro simulated saliva, gastric, and intestinal digestion followed by faecal fermentation reveals a potential modulatory activity of Epimedium on human gut microbiota. J. Pharm. Biomed. Anal..

[52] Xu B., Qin W., Chen Y., Huang J., Ma L., Yan X. (2025). Dietary Short-Chain fatty acids supplementation improves reproductive performance and gut microbiota in gilts. J. Nutr..

